# Genotype and Gene Expression Associations with Immune Function in *Drosophila*

**DOI:** 10.1371/journal.pgen.1000797

**Published:** 2010-01-08

**Authors:** Timothy B. Sackton, Brian P. Lazzaro, Andrew G. Clark

**Affiliations:** 1 Field of Ecology and Evolutionary Biology, Cornell University, Ithaca, New York, United States of America; 2 Department of Entomology, Cornell University, Ithaca, New York, United States of America; 3 Department of Molecular Biology and Genetics, Cornell University, Ithaca, New York, United States of America; University of California Davis, United States of America

## Abstract

It is now well established that natural populations of *Drosophila melanogaster* harbor substantial genetic variation associated with physiological measures of immune function. In no case, however, have intermediate measures of immune function, such as transcriptional activity of immune-related genes, been tested as mediators of phenotypic variation in immunity. In this study, we measured bacterial load sustained after infection of *D. melanogaster* with *Serratia marcescens*, *Providencia rettgeri*, *Enterococcus faecalis*, and *Lactococcus lactis* in a panel of 94 third-chromosome substitution lines. We also measured transcriptional levels of 329 immune-related genes eight hours after infection with *E. faecalis* and *S. marcescens* in lines from the phenotypic tails of the test panel. We genotyped the substitution lines at 137 polymorphic markers distributed across 25 genes in order to test for statistical associations among genotype, bacterial load, and transcriptional dynamics. We find that genetic polymorphisms in the pathogen recognition genes (and particularly in *PGRP-LC*, *GNBP1*, and *GNBP2*) are most significantly associated with variation in bacterial load. We also find that overall transcriptional induction of effector proteins is a significant predictor of bacterial load after infection with *E. faecalis*, and that a marker upstream of the recognition gene *PGRP-SD* is statistically associated with variation in both bacterial load and transcriptional induction of effector proteins. These results show that polymorphism in genes near the top of the immune system signaling cascade can have a disproportionate effect on organismal phenotype due to the amplification of minor effects through the cascade.

## Author Summary

Genetic variation for resistance to infection is widespread among insects and other organisms. However, the extent to which this variation in resistance is mediated by changes in infection-induced gene expression is not known. In this study, we assayed expression of immune system genes and bacterial load after infection in a genotyped panel of lines of the model insect *Drosophila melanogaster*. We find that statistical associations between genetic variants and bacterial load tend to cluster in genes encoding proteins involved in microbial recognition. Variation in suppression of bacterial growth is also determined in part by genetic variation in the expression of downstream components of the immune system that function to directly kill bacteria, despite finding no genetic variation in any single of these effector gene significantly associated with phenotype. Instead, it appears that activity differences in upstream components of the pathway have a cascading effect that results in larger variation in the expression of coordinately regulated downstream effector genes. These results imply that the interactions among genes need to be taken into account when assessing the phenotypic consequences of genetic variation, as signaling cascades such as those in the immune response have the potential to amplify the phenotypic effects of minor genetic variation in individual genes.

## Introduction

*Drosophila*, like other insects, use a generalized immune response to combat pathogens. Unlike vertebrates, the insect immune response consists solely of an innate response, with cellular and humoral (cell-free) arms [reviewed in 1]. Despite considerable knowledge of the molecular basis of the *Drosophila* immune response, and increasing understanding of the extent of natural genetic variation for immunocompetence in this system [2]–[4], relatively little is known about the role of network structure in shaping the phenotypic consequences of genetic variation.

Linking genetic variation in transcriptional regulation to differences in complex phenotypes has the potential to illuminate mechanistic aspects of genotype-phenotype associations. Passador-Gurgel and coworkers [5] identified several genes in which transcript levels significantly associate with survival times after exposure of *D. melanogaster* to nicotine. Other studies in *Drosophila* have identified transcriptional variation associated with male reproductive success [6], male body size [7], aggressive behavior [8] and locomotive behavior [9]. While in some cases it has been possible to show that genetically determined transcriptional differences are statistically correlated with phenotypic differences, these studies have generally not identified causal genetic variants. In *Drosophila*, linking genetic variation to phenotypic variation via transcriptional changes has proven difficult [10],[11]. The *Drosophila* immune system provides an ideal opportunity to examine the consequences of genetic variation and differences among lines in patterns of gene expression in the context of a well-defined network.

In *Drosophila*, the humoral response is initiated by the recognition of microbial cell wall component by proteins such as PGRPs and GNBPs [12]–[14]. These proteins activate two primary signaling pathways, the Toll and Imd pathways. The Toll pathway is primarily activated after infection by fungi and Gram-positive bacteria, whereas the Imd pathway is primarily activated after infection by Gram-negative bacteria [15],[16], although this specificity is not absolute [17],[18]. In addition to these primary signaling pathways, the JAK/STAT and JNK pathways are thought to play a role in immune response, largely as part of the general stress response and wound healing [19],[20]. Activation of the Toll and Imd signaling pathways leads to the translocation of NF-κB transcription factors (Relish, DIF, Dorsal) to the nucleus where they drive transcription of effector genes, which encode proteins that are directly involved in bacterial clearance, such as antimicrobial peptides. These effectors are then released into the hemolymph, where they act to directly kill invading microorganisms [21].

Previously, we have examined associations between bacterial load after infection with each of four different bacteria and genetic markers (SNPs and indels) in candidate genes on the *Drosophila melanogaster* second chromosome [2],[3]. Here, using markers in candidate genes on the third chromosome, we examine both bacterial load and gene expression phenotypes, testing associations between genotype, sustained bacterial load, and transcription level of approximately 400 known and putative immune system genes.

## Results

### Genetic variation for immune function on the third chromosome in *Drosophila*

**Figure 1:**
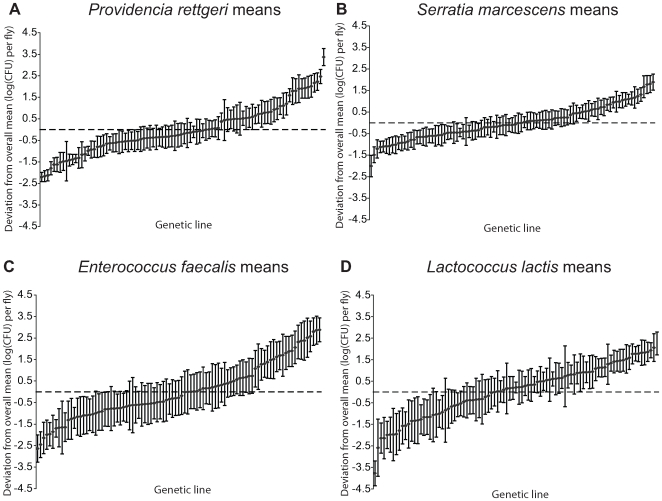
Mean bacterial load sustained 28 hours after infection by each of four different bacteria. Bacterial load is plotted as the deviation from the overall mean within each bacterium, adjusted for unbalanced data. The genetic lines are plotted independently in ascending rank order for each panel, and are not in the same order across panels. (A) *Providencia rettgeri* bacterial load. (B) *Serratia marcescens* bacterial load. (C) *Enterococcus faecalis* bacterial load. (D) *Lactococcus lactis* bacterial load.

We examined a sample of 94 third-chromosome substitution lines for variation in bacterial load sustained 28 hours after infection with each of four different bacteria: *Serratia marcescens*, *Providencia rettgeri*, *Enterococcus faecalis*, and *Lactococcus lactis* (Figure 1). In order to assess the effect of different third chromosomes on bacterial load phenotypes, we compared the likelihood of the data under a statistical model that includes variation among genetic (third-chromosome) lines as a main effect to the likelihood of the data under a model that does not. Likelihood ratio tests reveal a large, highly significant effect of third chromosome line on phenotypic variation in bacterial load against all four bacteria (*S. marcescens*: χ^2^ = 128.42, d.f. = 1, *P*<2.2×10^-16^; *P. rettgeri*: χ^2^ = 263.88, d.f. = 1, *P*<2.2×10^−16^; *E. faecalis*: χ^2^ = 51.533, d.f. = 1, *P* = 7.04×10^−13^; *L. lactis*: χ^2^ = 35.391, d.f. = 1, *P* = 2.70×10^−9^). Genetic line explains 66.9% of the non-error variance (14.5% of the overall variance) for load sustained after *S. marcescens* infection and 58.3% (22.1%) for load sustained after *P. rettgeri* infection, but only 27.4% (7.2%) for *E. faecalis* and 19.5% (6.2%) for *L. lactis* (Table 1). Total variance in bacterial load is much higher for the two Gram-positive bacteria (*E. faecalis* and *L. lactis*), as is residual variance and the fraction of total variance explained by experimental factors, suggesting that these infections produce noisier data (Table 1). The smaller fraction of variance attributable to line after infection with these two bacteria presumably stems from stochastic events during initiation and establishment of infection. The overall mean load sustained after infection also varies among bacteria, ranging from a low of 2,186 colony forming units (CFU) per fly 28 hours after infection with *S. marcescens* to a high of 653,436 CFU per fly after infection with *L. lactis*. Correlations of line means between bacteria (measured as Spearman's ρ) are generally moderate and positive (Table 2). While the positive sign of correlations between bacteria suggests that some genetic lines may have generally better immune responses, the relatively small magnitude suggests substantial bacteria-specific effects.

**Table 1:** Sources of variance in bacterial load phenotypes.

	Line	Experimental	Residual
*S. marcescens*	0.436 (0.145)	0.216 (0.072)	2.359 (0.783)
*P. rettgeri*	1.158 (0.221)	0.830 (0.158)	3.257 (0.621)
*L. lactis*	0.574 (0.062)	2.362 (0.256)	6.287 (0.685)
*E. faecalis*	0.876 (0.072)	2.323 (0.190)	8.998 (0.738)

Variance (fraction of total) attributable to the effect of genetic line, to experimental factors (infector, plater, day), and residual.

**Table 2:** Correlations among bacterial load phenotypes.

	*S. marcescens*	*P. rettgeri*	*L. lactis*	*E. faecalis*
*S. marcescens*		0.1369	0.02828	0.02527
*P. rettgeri*	+0.1561888		0.04021	0.00195
*L. lactis*	+0.2290430	+0.2145092		0.03012
*E. faecalis*	+0.2335280	+0.3202071	+0.2265	

Bottom diagonal: Spearman's rho; Top diagonal: *P*-value. Correlations were calculated using the function cor.test in R 2.6.0.

### Genotype-phenotype associations

We tested for statistical associations between bacterial load and genotypes at 137 polymorphisms in 26 genes and gene families on the third chromosome with known or suspected immune function. These included 6 antimicrobial peptide loci, 10 putative recognition loci (*GNBP*s and *PGRP*s), 8 known signaling loci, the Toll-like receptor Toll-9, and the iron-binding protein Transferrin 2 (Table 3). Our association test is based on mixed linear models: we assessed significance by comparing the observed model coefficient (effect size) for the marker in question to a null distribution generated from 5070 permuted data sets where phenotypes are randomly shuffled across lines while preserving linkage disequilibrium among genetic markers and correlations among bacterial loads after infection with different bacteria (see Methods for details of the permutation protocol).

**Table 3:** Candidate loci genotyped in this study.

Functional Class	Locus	Cytological Position	Markers typed
Antimicrobial peptide	*Attacin D*	90B6	2
Antimicrobial peptide	*CecAB*	99E2	4
Antimicrobial peptide	*CecC*	99E2	3
Antimicrobial peptide	*Drs*	63D2	2
Antimicrobial peptide	*DrsL*	63D1–2	4
Antimicrobial peptide	*dro2-5*	63D1	3
Recognition	*GNBP3*	66E5	5
Recognition	*GNBP1/GNBP2*	75D6	10
Recognition	*PGRP-LA*	67B1	7
Recognition	*PGRP-LB*	86E6	6
Recognition	*PGRP-LC*	67B1	10
Recognition	*PGRP-LD*	64E7–8	6
Recognition	*PGRP-LF*	67B1	4
Recognition	*PGRP-SB1*	73C1	5
Recognition	*PGRP-SB2*	73C1	6
Recognition	*PGRP-SD*	66A8	5
Signal transduction	*BG4*	94A1	5
Signal transduction	*ECSIT*	83C5	4
Signal transduction	*Rel*	85C3	6
Signal transduction	*Toll*	97D2	8
Signal transduction	*ird5*	89B1	5
Signal transduction	*pll*	97E11	5
Signal transduction	*spz*	97E1	7
Signal transduction	*tub*	82A5	2
Iron binding	*Tsf2*	69C4–5	4
Toll-like	*Toll-9*	77B6	9

We also tested for associations between SNP markers and the first principal component estimated from line means of bacteria after infection with each of the four different bacteria. This principal component is significantly positively correlated with load after infection with all four bacteria, suggesting that it represents a measure of general immune competence and/or general vigor. Results from this analysis recover statistical associations with markers that show significant associations with bacterial load measured after infection with multiple different bacteria, but do not uncover any additional general immune factors, and are not discussed further (Table S1). All statistical tests were implemented in R, as described in the Methods, and presented in Table S1.

Across all bacteria, 43 tests (7.85%) are significant at a nominal αof 0.05, and 12 tests (2.19%) are significant at a nominal α of 0.01; in both cases, we observe a significant excess of significant tests (α = 0.05: χ^2^ = 9.35, d. f. = 1, *P*-value = 0.0022; α = 0.01: χ^2^ = 7.84, d. f. = 1, *P*-value = 0.0051). Because some SNPs are in linkage disequilibrium and because bacterial loads across different pathogen challenges are weakly positively correlated, the 548 tests we conducted (137 markers by 4 phenotypes) are not likely to be independent. Thus, we also calculated the null distribution of significant SNPs based on permutations that preserve the correlation structure in the data (see Methods for additional details). We observe a mean of 28.6 significant tests under the null hypothesis at an α of 0.05, and a mean of 5.9 significant tests under the null hypothesis at an α of 0.01. In both cases, the number of significant tests we observe in the permuted data are significantly fewer than the values we observe in the real data (α = 0.05: 43 observed significant tests, *P*-value = 0.0323; α = 0.01: 12 observed significant tests, *P*-value = 0.0296).

Several markers in our dataset (8 and 2 at a nominal α of 0.05 and 0.01, respectively) are nominally associated with variation in multiple independent bacterial load phenotypes. Assuming all tests are independent, it is extremely unlikely that we would observe this number of SNPs associated with more than one bacterial load phenotype (α = 0.05: χ^2^ = 19.52, *P*-value (by simulation) = 0.00087; α = 0.01: χ^2^ = 45.42, *P*-value (by simulation) = 0.00299). To verify this conclusion in the face of non-independence among tests, we used a permutation approach to estimate the null distribution of the number of SNPs with two or more significant tests under the assumption of no genotype-phenotype associations (α = 0.05: *P*-value = 0.0118; α = 0.01: *P*-value = 0.0053; see Methods for details).

Significant tests at a nominal α of 0.01 (0.05) are not randomly distributed among bacteria: 83.3% (67.4%) of the significant cases represent associations between genotype and bacterial load after infection with Gram-negative bacteria (*S. marcescens* and *P. rettgeri*). Gram-positive bacterial load has higher residual error variance and higher experimental variance in our experiments (Table 1), which could lead to reduced power to detect associations with this phenotype. In order to test this hypothesis, we calculated power by simulation, assuming variances estimated from either the Gram-negative or Gram-positive bacteria in our study (see Methods for details). Although power is lower for our simulated Gram-positive data across a range of effect sizes and two assumptions about minor allele frequencies (Figure S1), if average effect size of associations is equal between the two bacterial types we would not expect to see such a substantial excess of Gram-negative associations. It is possible that the observed excess of associations with resistance to Gram-negative infection could be driven by a biological difference in the response of *D. melanogaster* to the specific Gram-negative and Gram-positive bacteria we employed in this study that results in less among-line variation in load after infection with these particular Gram-positive bacteria.

**Figure 2:**
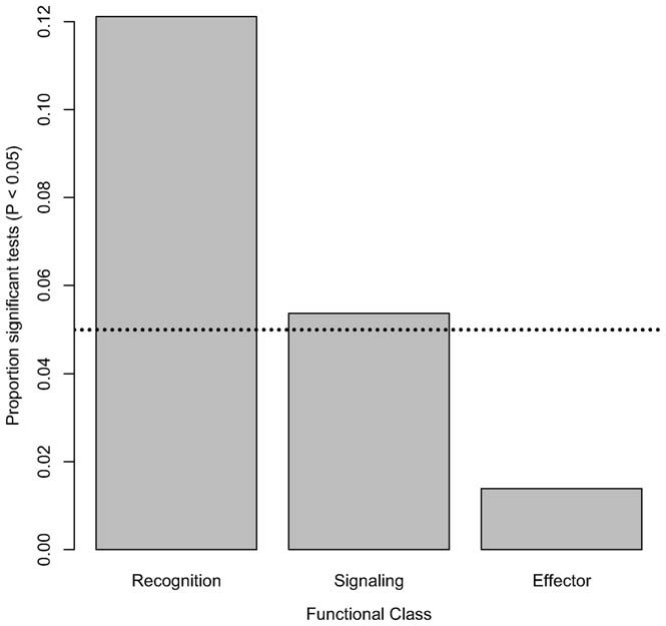
Distribution of significant associations among functional classes. Bar plot shows the proportion of tested markers within each functional class (pooled across all bacteria) that are significantly associated with bacterial load phenotypes at a nominal α of 0.05 (dotted line). The difference among classes is significant (χ^2^ = 11.35, d. f. = 2, *P* = 0.0034).

Nominally significant associations are also not evenly distributed within functional classes of the immune system. The proportion of tested markers that are associated with bacterial load phenotypes (at a nominal α of 0.05) significantly varies among functional classes (Figure 2; χ^2^ = 11.35, d. f. = 2, *P* = 0.0034). Markers in genes encoding recognition proteins have the highest proportion of significant associations with bacterial load (12.11% of tested markers in these genes are significantly associated with phenotype), followed by markers in genes encoding signaling proteins (5.36% of tested markers in these genes are significantly associated with phenotype). Markers in genes encoding effector proteins are rarely associated with differences in bacterial load (only 1.39% of tested markers in genes encoding effectors are significantly associated with phenotype). Average intralocus linkage disequilibrium is not significantly different among functional classes (data not shown), suggesting that this pattern is not driven by biases introduced by LD among SNPs. However, in order to rule out this possibility we generated a distribution for the fraction of significant associations in each of the three functional categories under the null hypothesis that there is no association between genotype and phenotype. Markers in genes encoding recognition proteins are significantly more likely to have significant associations (α = 0.05: 31 observed significant tests compared to a mean of 13.3 in the permutated data, *P* = 0.0016; α = 0.01: 10 observed vs. mean of 2.73 in permuted data, *P* = 0.0059). The same pattern does not hold, however, for markers in genes encoding signaling or effector proteins (α = 0.05: *P*_signaling_ = 0.492, *P*_effector_ = 0.965; α = 0.01: *P*_signaling_ = 0.500, *P*_effector_ = 1). Furthermore, while the average fraction of markers with significant associations at α = 0.05 (0.01) that are in recognition genes in the permuted dataset is 51.21% (50.96%), in the observed data it is 75.6% (83.3%).

**Figure 3:**
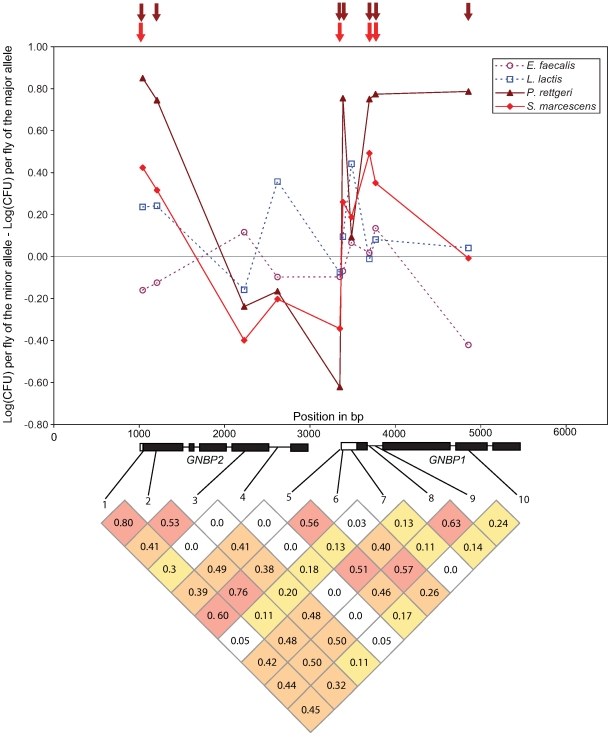
Genotype-phenotype associations at the *GNBP* locus in chromosomal band 75D. Upper panel: Plot of the effect size for each of the 10 SNPs genotyped at this locus. Gram-negative bacteria are shown in dashed lines, Gram-positive as solid lines. Arrows above the main graph indicate significance at a nominal α of 0.05. Lower panel: Pattern of linkage disequilibrium among the 10 genotyped SNPs. Grid shows R^2^ values, shaded by value: >0.50 red, 0.25-0.50 orange, 0.10–0.25 yellow.

Polymorphism at the GNBP75D locus, consisting of the genes *GNBP1* and *GNBP2*, is particularly striking in the extent and significance of associations with resistance to Gram-negative bacteria (Figure 3). Seven of the 10 SNPs at this locus are nominally significantly associated with variation in bacterial load after infection with *P. rettgeri*, although average linkage disequilibrium is high at this locus (average pairwise *r* ^2^ = 0.303; average pairwise D′ = 0.636). Four of those seven SNPs are also significantly associated with differences in bacterial load after infection with *S. marcescens*. These include one SNP in the 3′ UTR of *GNBP2* (GNBP75D_1041), one SNP in the 5′ UTR of *GNBP1* (GNBP75D_3350), and a pair of SNPs in the first intron of *GNBP1* (GNBP75D_3696 and GNBP75D_3768). Notably, GNBP75D_3696 is one of two SNPs that is significantly associated with differences in bacterial load after infection with two different bacteria at a nominal α of 0.01.

The haplotype structure at the GNBP75D locus is unusual for *D. melanogaster*. Despite spanning more than 2 kb, the four SNPs mentioned previously are found in only 6 of the 16 possible haplotypes in 91 of the 94 genetic lines (the remaining three lines have unique haplotypes). There are two major haplotypes (A-A-A-T and C-G-G-A) at frequencies of 0.244 and 0.449 respectively. When the phenotypes of the lines that carry these two haplotypes are compared directly using nonparametric tests, the A-A-A-T haplotype has a significantly higher median bacterial load after infection with both *S. marcescens* (median_AAAT_ = 8.12, median_CGGA_ = 7.48, Mann-Whitney U *P* = 0.036) and *P. rettgeri* (median_AAAT_ = 13.16, median_CGGA_ = 12.26, Mann-Whitney U *P* = 0.000299).

Further support for a biologically meaningful association of genetic differences at the GNBP75D locus with phenotypic variation comes from an analysis of per-gene significance. Because of the high intra-locus LD, we applied permutation tests to assess significance of effects attributable to genetic variation at each gene. We generated a null distribution of the sum of the χ^2^ test statistics for each marker within a locus under the assumption of no association between genotype and phenotype, while controlling for confounding effects of correlation among markers within a locus, and compared the observed sum of the χ^2^ test statistics to this permuted distribution (see Methods for details; full results in Table S2). We find significant evidence for an association between markers in GNBP75D and bacterial load after infection with *P. rettgeri* (*P* = 0.00059; *P* = 0.015 after multiple test correction using the Holm method implemented in the R function p.adjust) and, more weakly, *S. marcescens* (*P* = 0.023; *P* = 0.577 after multiple test correction as above).

*PGRP-LC* is another recognition gene with repeatable evidence for a significant association between SNPs and bacterial load, albeit somewhat weaker than the evidence for the GNBP75D association. In this case, genotypes at two out of 10 SNPs are associated with variation in bacterial load against at least two different bacteria at a nominal α of 0.05, and a third marker has marginal significance. A SNP marker approximately 125 bp upstream of the transcriptional start site of *PGRP-LC* (PGRPLC_884) is associated with resistance against both *E. faecalis* (*P* = 0.0556) and *S. marcescens* (*P* = 0.038). A SNP marker in the third exon of splice variant *PGRP-LC-RB* (PGRPLC_5624; intronic in splice variants *PGRP-LC-RA* and *PGRP-LC-RC*) is associated with variation in bacterial load against *L. lactis* (*P* = 0.0481) and *S. marcescens* (*P* = 0.006), with the same allele associated with lower bacterial load against both bacteria. Another SNP marker in the fourth exon (in the PGRP domain) of *PGRP-LC-RA* (PGRPLC_6635; in the intron of *PGRP-LC-RA* and *PGRP-LC-RC*) is also associated with variation in bacterial load against *L. lactis* (*P* = 0.0075) and *S. marcescens* (*P* = 0.0095). These two SNP markers are in linkage disequilibrium (*r^2^* = 0.193, *P* = 7.92×10^−4^), but neither is in significant linkage disequilibrium with the upstream marker.

### Marker by sex interactions

Empirical and theoretical work [22]–[24] suggests that immune function may differ between the sexes, as males and females make different resource allocation decisions between immune defense and reproductive output. These observations lead to the hypothesis that the genetic basis of the immune response may depend on sex: indeed, these kinds of genotype by sex interactions have been observed for other quantitative traits in *D. melanogaster* [e.g., 25]. To test this hypothesis, we compared the likelihood of our observed bacterial load data under a model with a Sex by Marker interaction to the likelihood of the data under a model without such an effect (but retaining the main effects of Sex and Marker). To assess the significance of the resulting likelihood ratio test statistics, we used a null distribution of likelihood ratio test statistics calculated by permuting the data 1000 times.

We find little evidence for significant effects of marker by sex interactions on bacterial load. While 6.93% of tests are significant at a nominal α of 0.05, a weakly significant excess over the null expectation (χ^2^ = 4.32, d. f. = 1, *P* = 0.0377), only 0.91% of tests are significant at a nominal α of 0.01, which is not different from the null expectation (χ^2^ = 0.042, d. f. = 1, *P* = 0.8367). While it is possible that there are weak marker by sex interactions that we do not have the power to detect in this experiment, we believe that such effects are likely to be small compared to main effects of SNP across sexes. There is a clear main effect of sex: males have consistently lower bacterial loads irrespective of genotype, consistent with the hypothesis that the sex bias in immune function is phenotypically plastic in *Drosophila*, and depends on food and mate availability [22]. We have only examined variation on the third chromosome in this study; a similar studies of genes on the second chromosome also find little evidence for substantial sex by SNP interactions [2],[3]. However, a recent study of variation in X-linked immune genes suggests substantial sex by SNP interactions [26].

### Measuring gene expression using BeadChips

In order to understand the mechanistic basis of differences in immune phenotypes linked to genetic variation on the third chromosome, we measured gene expression of approximately 700 transcripts in males from a subset of 30 of the 94 phenotyped chromosome 3 substitution lines. Using custom-designed Illumina BeadChip microarrays, we measured transcript abundance under three different conditions (uninfected, 8 hours post *S. marcescens* infection [Sm-infected], and 8 hours post *E. faecalis* infection [Ef-infected], where *S. marcescens* and *E. faecalis* were chosen arbitrarily to represent Gram-negative and Gram-positive bacteria respectively). We selected the subset of assayed lines to be biased toward the tails of the phenotypic distribution in order to enhance our power to detect correlations between transcript abundance and phenotype. We normalized and log-transformed expression values as described in the Methods. For most analyses, we focused on the Ef-induced (Ef-infected minus uninfected) and Sm-induced (Sm-infected minus uninfected) samples.

In addition to quantifying the 329 genes with a known or putative immune function (including 172 genes with some characterized function and 157 genes predicted to have a role in immunity based on transcriptional induction after infection), our BeadChip microarrays include genes involved in metabolism (139) and sex/reproduction (164), as well as 69 probesets consisting of housekeeping gene controls, and genes involved in insecticide resistance. Full details of the BeadChip design are described in the Methods; the full list of genes are presented as Table S3 (probe sequences are available upon request from T. B. S.). For most analyses, we focus on the 329 immune genes on the BeadChips, although in some cases we use the other genes as controls.

### Genotype-expression associations

Although with only 30 lines applied to the BeadChip arrays we have limited power to detect associations between SNPs and gene expression variation, we tested for significant associations by comparing a mixed model with a fixed effect of SNP to one with just a fixed intercept. Because permutations are not computationally feasible for the large number of tests required for this analysis, we assessed significance by comparing the likelihood ratio test statistic to a standard χ^2^ distribution. Overall, 3.55% (9.09%) and 2.98% (10.33%) of genotype-expression association tests are significant at a nominal α of 0.01 (0.05) in the Sm-induced and Ef-induced samples, respectively. In all cases it is highly improbable to obtain this many significant tests purely by chance under the assumption that regulation of expression of all genes is independent (Sm-induced, α = 0.01: χ^2^ = 6351, *P*<2.2×10^−16^; Ef-induced, α = 0.01: χ^2^ = 3833, *P*<2.2×10^−16^; Sm-induced, α = 0.05: χ^2^ = 5791, *P*<2.2×10^−16^; Ef-induced, α = 0.05: χ^2^ = 3416, *P*<2.2×10^−16^). The same pattern holds if we consider the absolute expression level in the Ef-infected, Sm-infected, and uninfected samples individually (data not shown).

Because we assumed the null distribution of the test statistic follows an asymptotic chi-square distribution, it is possible that the excess of significant *P*-values we observe is primarily due to mis-specification of the null distribution. We expect that polymorphisms in genes known to have a role in the immune system will be more likely to affect expression of immune-related genes than expression of other genes on the BeadChip. Indeed, for the Ef-induced sample, we see significantly more tests with both *P*<0.01 and *P*<0.05 among immune-related genes than other genes (P<0.01: 0.0325 vs. 0.0274; χ^2^ = 21.6874, d.f. = 1, *P* = 3.206×10^−6^; P<0.05: 0.0933 vs. 0.0889; χ^2^ = 5.6409, d.f. = 1, *P* = 0.01755), although this is not the case for the Sm-induced sample (but note that “non-immune” genes may still be responding transcriptionally to infection). Thus, while it appears that some of the genotyped SNPs in this study have significant effects on gene expression, particularly for the Ef-induced sample, limiting our experiment to 30 lines reduces our power to detect significant associations. Nonetheless, there are 304 and 350 associations between genotypes and induction of immune genes after *E. faecalis* and *S. marcescens* infection respectively significant at a 10% false-discovery-rate, which are presented in Table S4. Of particular note is the marker PGRPSD_494, which is associated with expression of 73 of the 329 immune genes we assayed. However, given the uncertainty in the true estimates of significance, we focus on overall qualitative patterns of genotype-expression associations.

### Significant associations tend to follow the predicted network structure

Because a considerable amount is known about the transcriptional feedback relationships in innate immune networks, we can make some predictions about the expected direction of associations between genotypes and variation in gene expression of specific genes. Most generally, we expect that markers in upstream genes in the immune pathway should predict expression of downstream genes much more often than vice versa. For example, we believe that genetic differences in signaling genes could lead to differential expression of effector genes, but that genetic differences in effector genes do not result in feedback that influences transcription of signaling genes. For both the Ef-induced and Sm-induced samples, we consistently see an excess of associations between markers in upstream loci and gene expression of downstream loci relative to associations between markers in downstream loci and expression of upstream loci (Table 4). This pattern is consistently more significant between “adjacent” functional classes in the immune network, although the recognition/effector pair is also the case with the smallest number of tests and thus the lowest power.

**Table 4:** Significant genotype-expression associations follow network structure.

		Ef-induced		Sm-induced	
Upstream Functional Class	Downstream Functional Class	Odds Ratio	P-value	Odds Ratio	P-value
Signaling	Effectors	1.939	0.0001	1.625	0.0056
Recognition	Effectors	1.337	0.2188	1.637	0.0631
Recognition	Signaling	1.566	0.0258	1.706	0.0084

Odds ratio represents the proportion of significant associations between upstream SNPs and downstream transcription relative to the proportion of significant associations between downstream SNPs and upstream transcription.

The network structure argument also has implications for the distribution of *cis* and *trans* associations across expression of effector, signaling and recognition genes. Specifically, while there is no reason to believe that *cis* associations should be related to network structure, we hypothesize that downstream categories (particularly effector genes) will have significantly more *trans* associations than upstream categories. For both Ef-induced and Sm-induced samples, we find support for this hypothesis. In the Sm-induced sample, 4.32% of tests between *trans* markers and expression of effector genes are significant, compared to 2.75% for expression of signaling genes and 2.37% for expression of recognition genes (χ^2^ = 47.6607, d.f. = 2, *P*-value = 4.473×10^−11^). In the Ef-induced sample, 4.64% of tests between *trans* markers and expression of effector genes are significant, compared to 2.65% for signaling genes and 2.62% for recognition genes (χ^2^ = 64.5568, d.f. = 2, *P*-value = 9.587×10^−15^). These differences remain significant if *trans* tests are split into those that involve markers in the same functional class as the expression phenotype being measured and those that involve markers in different functional classes (data not shown). In neither case do we observe significant differences in the proportions of *cis* tests that are significantly associated with gene expression phenotypes (data not shown), although pooled across all markers we observe a higher proportion of significant *cis* tests that *trans* tests (Ef-induced: Fisher's Exact Test *P* = 0.02737, Odds Ratio = 1.99; Sm-induced: Fisher's Exact Test *P* = 0.08566, Odds Ratio = 1.69).

To dissect the role of crosstalk and cross-regulation between signaling pathways in the pattern of associations between gene expression and SNPs, we examined the number of significant associations between markers in signaling genes in either the Toll or Imd pathway and expression of signaling genes in other signaling pathways. On the BeadChips, we have representatives from the Toll, Imd, JAK/STAT, JNK, Ras, p38, and Notch signaling pathways. We compared the observed number of tests significant at α = 0.01 (excluding potential *cis* associations) to the expected number based on chance alone, using χ^2^ tests. For the Ef-induced sample, we observe a significant excess (over chance expectations) of associations between markers in signaling genes in the Toll pathway and induction of signaling genes in the Toll pathway (*P* = 1.32×10^−13^) and the JAK/STAT pathway (*P* = 3.05×10^−14^); we also observe an excess of significant associations between markers in signaling genes in the Imd pathway and induction of signaling genes in the Imd pathway (*P* = 0.00159) and the Toll pathway (*P* = 0.0292), although the latter is not significant after Bonferroni correction.

For the Sm-induced sample, we see a similar pattern. There is a significant excess of significant associations between markers in signaling genes in the Toll pathway and induction of signaling genes in the Toll pathway (*P* = 1.32×10^−13^), and to a lesser extent induction of signaling genes in the Imd pathway (*P* = 0.0341) and the JAK/STAT pathway (0.0496), although the latter two *P*-values do not survive a Bonferroni correction. Markers in signaling genes in the Imd pathway are significantly more likely than expected by chance to be associated with induction of signaling genes in the Imd pathway (*P* = 0.0219) and the JAK/STAT pathway (*P* = 0.00102) after infection with *S. marcescens*.

Because the numbers of markers in signaling genes represent a relatively limited sample, some caution should be used in interpreting these results. Nonetheless, these data suggest that, in addition to self-regulation of both the Toll and Imd signaling pathways by components of the pathway, there is some crosstalk between the Toll, Imd, and JAK/STAT pathways, although there seems to be relatively little crosstalk between either of the Toll or Imd pathways and the JNK pathway, at least at the time point we examined (8 hours after infection). Given genetic variation for flux through the pathway, these patterns of autoregulation and cross-regulation may have the effect of amplifying the phenotypic consequences of minor genetic variants.

### Quantitative trait transcripts

Considerable recent interest has focused on identifying not just genetic markers that associate with quantitative variation in phenotypes, but also transcripts whose abundance correlates with phenotypes of interest [5],[10],[27]. These attempts have had mixed success, with some studies failing to find any significant correlations between transcript abundance and phenotype [e.g., 10] and others finding some evidence for significant associations [e.g., 5].

**Figure 4:**
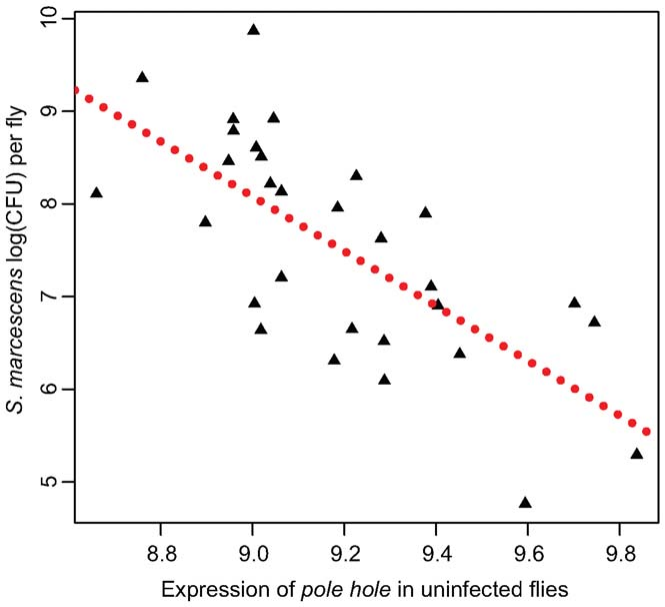
Expression of *pole hole* correlates with *S. marcescens* bacterial load. Normalized expression of *pole hole* in uninfected flies plotted against bacterial load 28 hours after infection with *S. marcescens*. See Methods for details.

Here, we used a simple regression of the induction of immune-related transcripts against either *E. faecalis* bacterial load (for Ef-induced sample), *S. marcescens* bacterial load (for Sm-induced sample), or overall bacterial load (as measured by the first principal component from all four bacterial load measures) to attempt to detect expression-phenotype associations. In this analysis, induction correlates with bacterial load for very few transcripts. Only the induction of *Attacin C* and *Drosocin* after *E. faecalis* infection correlate with *E. faecalis* bacterial load at a false discovery rate of 10%. Induction levels after *S. marcescens* infection do not appear to correlate with *S. marcescens* load for any transcripts, although uninfected expression level of *pole hole* (D-Raf) associates with *S. marcescens* load at a FDR of 0.0035, the most significant transcriptional association in our dataset (Figure 4). Uninfected transcriptional levels of six genes (*CG30088*, *phl*, *Thor*, *Keap1*, *Dif*, *IM1*) significantly associate with a principal component measuring overall immune competence and/or general vigor, at an FDR of <10%. Interestingly, *pole hole* is necessary for the proliferation or survival of circulating hemocytes in *D. melanogaster* [28],[29] suggesting that flies with lower levels of *phl* transcription may have fewer hemocytes and will be less able to resist infection.

Our analysis suggests that naturally occurring variation in expression level of individual genes, measured as either induction after infection or as absolute expression in uninfected flies, is a weak predictor of bacterial load phenotypes. This result suggests that, unlike complete or nearly complete knockdowns of single genes, which can have dramatic effects on bacterial load, the differences in expression of immune genes among lines that is observed in natural populations has relatively subtle consequences. However, given the structure of the immune network, this observation may not be surprising. The immune system is a highly co-regulated system, in which small changes in expression of upstream components can be amplified among downstream genes, and multiple feedback loops provide for some measure of self-regulation of the system. Furthermore, correlated transcription of many effectors could indicate that the overall extent to which the immune system (in whole or in part) is transcriptionally activated after infection is more biologically relevant than variable levels of activation of any one gene. In order to test this hypothesis, we considered whether principal components obtained from the correlation matrix among transcriptional profiles of subsets of genes predict phenotype. As an added advantage, the method of principal components reduces the dimensionality of large datasets, improving power.

### Principal component analysis

Our initial hypothesis is that the most important transcriptional determinant of phenotype is the extent to which effector proteins are induced after infection. To measure this, we initially constructed a set of principal components (PCs) from the 61 genes in our dataset with a known or putative “effector” function. These include antimicrobial peptides, components of the phenoloxidase cascade, lysozymes, putative iron-sequestration proteins, and some less-well-characterized genes such as the Turandots. For both the Sm-induced dataset and the Ef-induced dataset, the variance explained by the first principal component is substantially higher than the variance explained by any other, and so we have focused on the first PC when looking for correlations with phenotypes.

**Figure 5:**
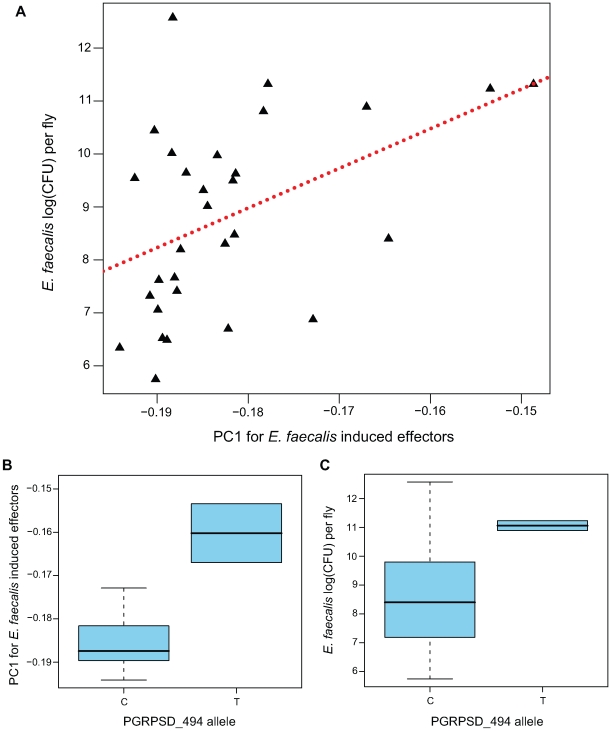
Genotype→gene expression→phenotype associations between PGRPSD_494 allele, Ef-induced expression of effectors, and *E. faecalis* bacterial load. (A) First principal component from the expression of effectors in the Ef-induced sample, plotted against bacterial load 28 hours after *E. faecalis* infection. (B) Box plot of the first principle component from the expression of effectors in the Ef-induced sample for the two allelic states at the PGRPSD_494 marker. (C) Box plot of bacterial load 28 hours after *E. faecalis* infection for the two allelic states at the PGRPSD_494 marker.

This first PC estimated from the effector genes in the Ef-induced sample is significantly positively correlated with *E. faecalis* bacterial load (Figure 5A; β = 74.8, F_1,28_ = 7.309, *P* = 0.01153), explaining just over 20% of the variance among lines in resistance to *E. faecalis* (*r^2^* = 0. 207). This PC is dominated by negative loadings of several antimicrobial peptide genes (*Mtk*, *DptB*, *AttC*, *Drs*) and genes encoding several uncharacterized peptides known to be induced by infection (*IM23*, *IM10*, *TotM*, *IM2*, *IM4*, *IM1*). The full set of loadings is available as Table S5. Thus, this analysis suggests that genetic lines that induce antimicrobial peptides (and potentially related peptides) more strongly (*i.e*., have a lower PC1) sustain a lower bacterial load and thus are better able to resist infection.

We also examined the Sm-induced sample using a similar procedure. However, we do not see any correlation between the first PC from the effector genes in the Sm-induced sample and *S. marcescens* bacterial load (*β* = 7.819, F_1,28_ = 0.2491, *P* = 0.6216), despite the fact that the Sm-induced PC is quite similar to the Ef-induced PC in terms of loadings. *Serratia marcescens* is resistant to the antimicrobial effects of Cecropins [30], Drosocins, and Defensins [31] suggesting that this bacterium may be particularly resistant to *Drosophila* antimicrobial defenses and providing a plausible hypothesis for the lack of effect of variation in effector gene induction on variation in bacterial load. The resistance of *S. marcescens* to antimicrobial peptides may also explain the disproportionate effect of expression level of the hematopoetic gene *pole hole* on resistance to *S. marcescens* infections, as cellular immunity may be the main mechanism of *D. melanogaster* resistance to *S. marcescens*.

### Genetic associations with PC1

A major challenge of quantitative genetics in *Drosophila* has been to link genetic polymorphisms to phenotypes via differences in expression. In this study, we have shown correlations between transcript abundance and phenotype, as well as correlations between genotype and phenotype. To look for genotype-expression-phenotype correlations, we focused on the *E. faecalis* bacterial load phenotype and the Ef-induced expression sample, and asked whether any of the SNPs that have nominally significant correlations with bacterial load are also correlated with the effector induction PC1. Of the eight SNPs with at least nominal associations between genotype and phenotype (*P*<0.05), we find that one of them, PGRPSD_494, is also statistically associated with effector induction PC1 (Figure 5B; *β* = 0.0235, *F*_1,27_ = 11.4, *P* = 0.002237), explaining nearly 30% of the variance in this principal component (*r* ^2^ = 0.297).

The PGRPSD_494 marker is a C/T polymorphism located approximately 500 bp upstream of the transcriptional start site of *PGRP-SD*. The T allele is associated with both a higher bacterial load after infection (Ef load_T-C_ = 0.6741; *P* = 0.02) and with lower induction of antimicrobial peptides (higher PC1; Figure 5C). *PGRP-SD* has been shown to have a role in the recognition of some Gram-positive bacteria, including *E. faecalis* [32], and our data suggest that naturally occurring variation in *PGRP-SD* may in fact mediate the strength of the transcriptional response to infection, and thus the ability of the fly to resist infection. This site does not appear to be significantly associated with induction or naïve expression of *PGRP-SD* in our data, but as mentioned previously it is associated with induction levels of 73 of the 329 immune genes we assayed. No other SNP in our dataset is associated with induction levels of more than 14 genes, and most are associated with induction levels of fewer than 10 genes.

### Functional differentiation of *PGRP-SD* alleles

**Figure 6:**
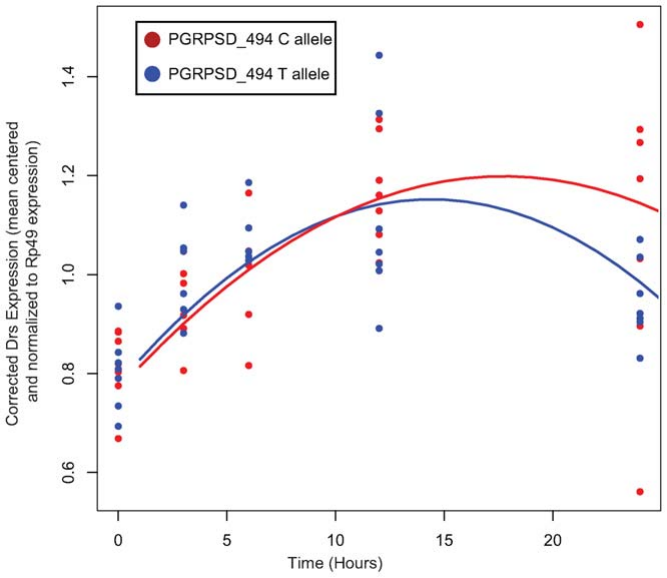
Normalized expression of *Drs* after infection with *E. faecalis*. Each of the two sampled alleles of *PGRP-SD* are plotted separately. There is a significant time by allele interaction (P = 0.01072, see Table 5 for details). Lines show the fitted model for each allele.

In order to test whether there is differential activation of either the Toll or the imd signaling pathway in lines carrying alternate alleles of PGRP-SD, we selected 7 random lines carrying the T allele and 7 random lines carrying the C allele at the PGRPSD_494 marker for further study. We infected these 14 lines with *E. faecalis* (as described in the Methods) and then, at five time points post-infection, assayed expression of two antimicrobial peptides that are commonly used as read-outs for the two major immune signaling pathways in *Drosophila*: *DptA* for the Imd pathway, and *Drs* for the Toll pathway. We find that there is a significant time by allele interaction for *Drs* expression (Table 5), but not *DptA* expression (data not shown), suggesting that the dynamics of Toll pathway activation are significantly different depending on which PGRP-SD allele a given fly line carries. Specifically, we find that lines carrying the PGRPSD_494 ‘C’ allele sustain Toll pathway activation at higher levels that those carrying the PGRPSD_494 ‘T’ allele (Figure 6), consistent with the observation that fly lines carrying the ‘C’ allele both have higher expression of effectors (measured by the effector PC1 described above) and sustain lower bacterial loads. Taken together, these results suggest that allelic state at PGRP-SD has a significant impact on downstream transcript abundance via modulation of Toll pathway activation dynamics, which in turn leads to observable differences in immune phenotypes.

**Table 5:** Fixed-effect terms of the linear model of *Drs* expression.

Term	Estimate	MCMC mean	*P*-value
**(Intercept)**	**+1.01187**	**+1.01195**	**0.00001**
**Time**	**+1.43119**	**+1.43512**	**0.00001**
**Time^2^**	**−1.07752**	**−1.07685**	**0.00002**
PGRP-SD allele	−0.02859	−0.02811	0.41114
Time * PGRP-SD allele	**−0.93596**	**−0.94389**	**0.01072**
Time^2^ * PGRP-SD allele	−0.33789	−0.34431	0.34616

Polynomial terms are orthogonal polynomials calculated using the R function poly(); p-values are estimated by MCMC (100,000 samples) using the R function pvals.fnc(). Significant terms are in bold.

## Discussion

The pursuit of an understanding of underlying determinants of phenotypic variation in *Drosophila* has a long history [33]. More recently, the availability of high-throughput gene expression microarrays has generated interest in correlating variation in transcript abundance across genetic lines with differences in phenotypes [5]–[7],[10],[11]. However, datasets that include both genotype information and transcriptional variation have been rare [but see 10],[34]. In this paper, we have focused on attempting to predict immunocompetence in *D. melanogaster* from SNPs in candidate genes and transcript abundance, guided by the known structure of the innate immune network [1].

The strong context dependence of association test results leads us to focus on trends across functional classes of genes instead of individual statistical associations between markers and bacterial load phenotypes. We take advantage of the replication of our experiment across four different bacterial strains, as well as patterns of nominally significant associations within genes, to increase confidence in our associations. We identify two loci, both encoding proteins involved in bacterial recognition, that appear to harbor genetic variation that is consistently associated with differences in bacterial load phenotypes.

One of the these loci contains the closely linked genes *GNBP1* and *GNBP2*. Several SNPs representing a single major haplotype are associated with differences in bacterial load after infection with both *S. marcescens* and *P. rettgeri*. It is somewhat unexpected to suggest a role for variation at *GNBP1* in resistance against Gram-negative bacteria, as *GNBP1* has only been shown to be involved recognizing Gram-positive bacteria and activating the Toll signaling pathway [35],[36]. However, the major haplotype spans both genes, making it impossible to determine the causal variant, and no definitive role for *GNBP2* is known. Notably, however, the statistical support for an association between variation at this locus and bacterial load after *P. rettgeri* infection is particularly strong, and is significant even after strictly controlling family-wise error rates. The second of these loci encodes *PGRP-LC*, the major receptor in the Imd pathway [12], [37]–[39]. Three SNPs at this locus are associated with differences in bacterial load after infection with *S. marcescens*, *E. faecalis*, and *L. lactis*. The observation that variation in the Imd pathway, canonically thought to be principally involved in resistance to Gram-negative bacteria, appears to associate with differences in bacterial load after infection with Gram-positive bacteria suggests that the innate immune network is dynamic with extensive feedback, co-activation and crosstalk, consistent with previous work demonstrating synergistic activation of the immune response by the Toll and Imd pathways in combination [40]. This pattern is further demonstrated by the pattern of associations between genotype and gene expression: there are significantly more associations than expected by chance between SNPs in both the Toll and Imd pathways and signaling genes outside those pathways (particularly in the JAK/STAT pathway).

This study, combined with previous candidate-gene-based association studies between immunocompetence and polymorphisms on the second chromosome [2],[3], allows us to infer general patterns about the genetic architecture of immunocompetence in *Drosophila*. Most of the significant associations between SNPs and phenotype that we observe in this study are in genes encoding recognition proteins, primarily *PGRP*s and *GNBP*s, suggesting that variation in upstream components of the signaling network has substantial phenotypic consequences. Strikingly, we find a near-complete lack of significant associations, even without correcting for multiple tests, in antimicrobial peptides. In this study and in the previous studies, we genotyped 204 markers covering every known antimicrobial peptide in *D. melanogaster*. Only a single marker (CecC_1660), a noncoding SNP downstream of *CecC*, has a nominal *P*-value less than 0.05, and even that marker is unlikely to be a true association, as the association neither survives multiple test correction nor is observed in multiple experiments. Taken together, these studies provide convincing evidence that any functional effect of genetic variation in *D. melanogaster* AMP genes is far too small to be observed in experiments such as these. This observation supports the previous inference from genetic evidence that *Drosophila* AMPs are at least partially functionally redundant [41].

A different picture is painted when considering the effect of variation across lines in overall transcript abundance. Here, the total induction of effector genes (primarily AMPs and other induced peptides such as the Turandots) appears to correlate with bacterial load, at least after *E. faecalis* infection. Together, these observations suggest that while *cis*-acting variation in individual AMPs may be of little consequence overall for resistance to bacterial infection in *D. melanogaster*, the combined output of AMPs after infection is a critical determinant of resistance. Thus, genetic polymorphisms that influence expression of many downstream components of the pathway can potentially have large effects on resistance phenotypes, as appears to be the case for the PGRPSD_494 marker. We additionally note that in both the present and in our previous studies, SNP associations in candidate genes have failed to explain the entirety of the observed genetic variance. This indicates genetic variation for immunocompetence that maps to genes outside our candidate list, or to more complex (epistatic) interactions among genes.

The combination of genetic polymorphism, bacterial load phenotypes, and transcript abundance thus allows us to propose a model of the genetic architecture of immunocompetence informed by the structure of the innate immune network. Genetic variation in genes encoding proteins at the top of the network (such as recognition proteins) can be amplified by the pathway (as demonstrated by the association between variation at *PGRP-SD* and the Ef-induced effector PC1), leading to more significant associations with phenotype. However, genetic variation in genes encoding proteins at the bottom of the network, such as AMPs, has relatively little effect, as changes in any single effector protein do not seem to cause large enough effects on phenotype to be detectable in experiments of the scale we have performed. Since there appears to be relatively little feedback between SNPs in effector proteins and transcription of upstream genes (as demonstrated by the dearth of associations between effector SNPs and signaling gene transcripts), these effector SNPs probably have relatively little impact in *trans*. Overall, then, it is polymorphisms in upstream genes, and especially recognition genes, that lead to variation in abundance of effectors, and ultimately to fitness differences among lines (to the extent that resistance to infection correlates with overall fitness), while single mutations in antimicrobial peptides are likely to be of relatively little consequence.

This view of the evolutionary and fitness consequences of mutations in different components of the immune response is consistent with what is known about the evolutionary history of immune system genes in *Drosophila*. Population genetic and molecular evolutionary studies have suggested little evidence for adaptive evolution in antimicrobial peptides [42]–[44], which might be expected given the lack of evidence for fitness consequences attributable to segregating variation in these genes reported in this study and others [2],[3]. In contrast, we see significant evidence for adaptive evolution in upstream components of the immune system [42],[43],[45]; it is these genes that appear to harbor the population variation with the largest consequences for individual fitness. By combining expression data, genetic data, and knowledge of network structure, we can gain a much better understanding of the phenotypic consequences of genetic variation than any one component could provide alone.

## Methods

### *Drosophila* lines and bacterial stocks

We evaluated ninety-four lines of *D. melanogaster* for resistance to infection against each of four different bacteria. These lines are originally derived from a natural collection of wild-caught *D. melanogaster* from State College, PA by Anthony Fiumera. Each line in the panel is homozygous for an individual third chromosome isolated from the natural population and substituted into a common genetic background. The construction of these lines is described in more detail in Fiumera *et al.* [46]. The third chromosome is the largest *D. melanogaster* chromosome, containing about 44% of the euchromatic genome, including genes encoding proteins from all major functional classes of the immune system, and thus represents the most natural chromosome on which to focus our study. The *D. melanogaster* lines in this study were challenged with each of four different bacteria, two Gram-positive and two Gram-negative. The Gram-positive bacteria used are the *E. faecalis* and *L. lactis* strains described in Lazzaro *et al.* [2]. The Gram-negative bacteria used are the *S. marcescens* strain described in Lazzaro *et al.* [2], and *Providencia rettgeri* [47].

### Survey sequencing and genotyping

We ascertained markers to be genotyped by sequencing the complete coding region and 1–2 kb upstream of 25 candidate loci (listed in Table 3) from 8 lines. We selected loci to represent genes encoding relatively well-characterized proteins that encompass a range of immune functions. While using a candidate gene approach necessarily means that we will not sample every polymorphism that may be associated with phenotypic differences among lines, our primary goal of capturing sufficient polymorphism to test hypotheses about the role of network structure mediating genotype-phenotype associations is well served by such an approach. We assembled sequencing reads into contigs using Sequencher and manually identified SNPs and indels to assay in the full panel of 94 lines. We used three different methods for genotyping our panel of lines. Approximately half of the markers were genotyped using SNPlex (Applied Biosystems, Foster City, CA) and the remaining markers were genotyped using pyrosequencing assays, SNPstream (Beckman Coulter, Fullerton, CA), or fRLFP [48]. A small number of markers were genotyped with both SNPlex and pyrosequencing; for the rare cases where the genotype call disagreed, we used the SNPlex call. After genotyping, SNPs were filtered to produce a set of 137 usable markers (136 SNPs and 1 indel): markers with a minor allele frequency <0.05 were dropped, and only one marker (chosen at random) was kept from any pair with LD (measured by r^2^)>0.90. Annotation information for each SNP, including the genotyping method used to assay each SNP in the 94 lines, are presented as Table S6. Filtered genotype calls for each line are presented in Table S7. Linkage disequilibrium between each pair of genotypes is provided in Table S8.

### Bacterial infections

We infected the 94 *D. melanogaster* lines in a complete-block design, with each line infected on each of three different days. On each day, each line was infected by one of 3 to 5 infectors at random, and a different infector infected each line on each day. Typically 2-3 replicates per line per sex were obtained on each day, for a total of 12–18 replicate data points for each *D. melanogaster* line. The entire experiment was repeated independently for each bacterial challenge. Flies were artificially infected by septic pinprick as described previously [2],[3]. Briefly, we pierced the thoraces of individual *D. melanogaster* aged 3–5 days post-eclosion with a 0.1-mm dissecting pin (Fine Science Tools, Foster City, CA) coated in liquid culture (OD600 = 1.0±0.2) of the bacterium of interest, delivering an average of 4×10^3^ bacteria to each fly. *Drosophila* were maintained at 22°–24°C on a rich dextrose medium for the duration of the experiment. To measure bacterial load, we homogenized same-sex trios of flies 28 hours post-infection in 500 µl of sterile LB and then quantitatively plated the homogenates on standard LB agar plays using robotic spiral platers manufactured by Spiral Biotech (Bethesda, MD) and Don Whitley Scientific (Fredrick, MD). We incubated the plates overnight at 37°C and then estimated the concentration of viable bacteria in each homogenate using the colony counting systems associated with each plater. Prior to plating, we diluted homogenates of *L. lactis* 1000-fold, homogenates of *P. rettgeri* 100-fold, and homogenates of *E. faecalis* 10-fold, all in sterile LB, in order to correct for anticipated high bacterial loads. Our estimates of bacterial load per fly were transformed to correct for these dilutions before analysis. Mean bacterial load sustained by each line against each of the four bacteria is presented in Figure 1 and Table S7.

For some analyses, we generated a principal component from bacteria load line means after infection by each of the four bacteria using the prcomp() function in R. This principal component is positively correlated with load after infection with all four bacteria, suggesting it represents a common measure of immunocompetence across bacteria. However, it is also likely that this principal component captures some aspects of general vigor.

A number of recent studies have suggested that bacterial load sustained after infection and survival to infections are not strongly correlated in *Drosophila melanogaster*, suggesting that survival may be mediated in part by tolerance to bacterial loads [49]–[51]. In this study, we focus on resistance, as defined by bacterial load sustained 28 hours after artificial infection. Although knowledge of the molecular mechanisms that determine tolerance is increasing [52], there is not yet sufficient understanding of the underlying mechanistic basis for tolerance phenotypes to allow fruitful candidate gene association studies or to develop models based on network structure and functional attributes of candidate genes.

### BeadChip design

We selected 329 immune genes for inclusion on the custom Illumina BeadChips based on a number of criteria, including evidence for transcriptional regulation by infection in previous microarray experiments, genetic or molecular evidence for a role in immunity, and homology to known immune proteins in *D. melanogaster* or other organisms. The remaining 384 non-immune genes were selected either as controls or for other experimental reasons. Each gene is represented by two different probes, each of which is represented by an average of 30 beads on the array, giving an extremely high degree of technical replication. Given the number of samples assayed (as described below), we determined that genome-wide expression approaches were not practical; however, since numerous previous studies in *D. melanogaster* have identified a robust set of immune-regulated transcripts [15],[20],[53] we believe that a targeted expression approach represented by custom Illumina BeadChips captures the vast majority of genes whose expression is regulated by infection.

### Expression infections

We selected a total of 30 lines for our expression analysis, biased towards the upper and lower tails of the phenotypic distribution. Males of each line were either infected with *S. marcescens* with *E. faecalis*, as described above, or left uninfected, and then frozen 8 hours after treatment. We chose to use an 8-hour post-infection timepoint as a compromise between earlier time points, where the transcriptional response to wounding could be confounding, and later time points that risked missing transcriptional events that would be relevant to bacterial load at 28 hours after infection. We extracted total RNA using Trizol (Invitrogen Corp., Carlsbad, CA) following standard protocols, then made cDNA and amplified RNA samples following the BeadChip protocol.

### BeadChip hybridizations and data normalization

RNA samples were hybridized to BeadChips following standard protocols and scanned. After scanning, we normalized the data using the qspline method in the beadarray package for R. Mean probability of detection and signal intensity of control genes were used as hybridization quality control: for samples that failed to pass quality control checks, cDNA synthesis, RNA amplification, and hybridization were repeated from the original RNA extractions. Normalized induction after *E. faecalis* and *S. marcescens* infection (where induction is measured as log_2_ signal intensity for the infected sample minus log_2_ signal intensity for the uninfected sample), as well as unnormalized expression data from all treatments (Ef-infected, Sm-infected, Naïve) are presented as Dataset S1 and Dataset S2, respectively.

### Quantitative PCR

For quantitative PCR experiments, we sampled three replicates of 5–7 flies from each of 14 lines (7 carrying the C allele at PGRP-SD_494, 7 carrying the T allele, randomly selected) at five time points: uninfected (0 hours), 3 hours post-infection (with *E. faecalis*), 6 hours post-infection, 12 hours post-infection, and 24 hours post-infection. Flies were frozen in liquid nitrogen, RNA was extracted with Trizol, first strand cDNA synthesis was carried out, and qPCR TaqMan assays were run using standard protocols. We measured expression of three different genes: Drs, DptA, and Rp49. TaqMan probe and primer sequences, and reaction conditions, are available upon request from T.B.S. Data points with raw Rp49 CT values more than 1.5 times the interquartile range from the median were removed to eliminate samples with very little RNA or poor reverse transcription efficiency. Raw 1/CT values were normalized by Rp49 expression and values for each plate were mean-centered. Normalized expression of either Drs (Toll pathway) or DptA (Imd pathway) was then used as the response variable in the following second-order linear model:
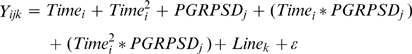
(1)where Y is normalized expression, Time (*i* = 0,3,6,12,24) is time after infection measured in hours, and PGRPSD (*j* = C, T) is allele at the PGRPSD_494 marker, and Line (*k* = 3F, 3E, 8A, 12E, 9D, 7C, 4C, 11F, 6E, 1C, 9E, 1E, 7D, 6H) is the genetic line and is treated as a random effect nested within PGRPSD. Because the response to time is not linear, we fitted a second-order model with a linear and quadratic time term, using the poly() function in R to estimate orthogonal polynomial terms.

### Statistical analysis

In order to test for associations between genotype and phenotype, we analyzed the following model using the package lme4 in R 2.6.0,
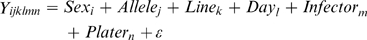
(2)where Y is bacterial load, Sex (*i* = 1,2) and Allele (*j* = 1,2) are main effects, and Line (*k* = 1,94), Day (*l* = 1,3), Infector (*m* = 1,5), and Plater (*n* = 1,2) are random effects. To assess significance, we compared the model coefficient for the Allele term to the null distribution obtained by permuting the genotype vector assigned to each line 5070 times and reanalyzing the data with the same model. The permutation approach was carried out as follows: for each row of the dataset, we have columns representing the four bacterial load phenotypes and the 137 genetic markers. For each permutation iteration, we randomize the phenotype vector with respect to the genotype vector, but do not shuffle relationships between among load phenotypes or among genetic markers. In this way, the permutation procedure preserves the correlations among bacterial loads and among genetic markers, but randomizes the association between genotype and phenotype.

For each permutation, we retain the estimated model coefficient (effect size), and the χ^2^ statistic for the test of the alternate and null (without an Allele term) model. Because the permutations shuffle the full genotype vector assigned to each line, rather than individual allele states, linkage relationships among markers are preserved in the permuted data. We use this fact to correct for linkage relationships among markers for many tests. Using the χ^2^ statistics from the permutated data, we can generate null distributions of *P*-values under the appropriate linkage conditions but assuming no significant genotype-phenotype associations.

We also use the χ^2^ statistics to estimate a combined probability of an association between all markers in a loci and a bacterial load phenotype. In this case, we sum the χ^2^ statistics for each marker in a loci for the permuted dataset, and use that distribution as a null distribution to compare the observed sum of χ^2^ statistics within each gene.

For our simulations to estimate the power of our experiment, we collapsed Day, Infector, and Plater terms into a single Experimental Error term, and then simulated 10,000 replicate datasets for each combination of Gram type (positive or negative), minor allele frequency (0.25 or 0.5) and Allele coefficient (0 to 1 in 0.1 increments). Each simulation assumes 3 replicates per experimental treatment (n = 3), per sex (n = 2), per line (n = 94), for a total of 18 data points per line and 1692 per simulation. This approximates our experimental conditions, with the caveat that the simulations assume no missing data and so will be an upper bound on our true power. Error terms are assumed to be normally distributed with a mean of 0 and variance equal to our estimated variance terms from Table 1, averaged across either Gram-positive or Gram-negative bacteria. To calculate power, we counted the number of tests significant at a nominal α of 0.01; significance was estimated by comparing the fit of a mixed linear model that included Line and Experimental Error as random effects and Allele and Sex as fixed effects to the fit of a null model that does not include a fixed effect of Allele.

In order to test for sex*marker interactions, we used a similar approach. In this case, we compared the likelihood of the data under the null model specified by equation (2) to likelihood of the data under the following alternative model:
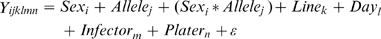
(3)where all terms are as described above. To assess significance, we compared the likelihood ratio test statistic obtaining by comparing the null and alternative models to the empirical null distribution of likelihood ratio test statistics obtained by analyzing 1000 permuted datasets in which the genotype vector assigned to each line was shuffled.

To test for associations between genotype and expression, we compared the likelihood of the data under the following linear model:
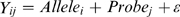
(4)where Y is the normalized induction of a given gene (where induction is measured as log_2_ normalized signal intensity for the infected sample minus log_2_ normalized signal intensity for the control sample), Probe (*j* = 1,2) is a random effect representing the two probes on the array for each gene, and Allele (*i* = 1,2) is the fixed main effect of interest, to the likelihood of the data under the null model that retains the random effect of Probe but includes only a fixed intercept. As the number of tests is far too large for permutations to be computationally feasible, we used the anova() function in lme4 to assess the significance of the alternative model using a likelihood ratio test.

In order to test for correlations between transcript abundance and phenotype, we used two approaches. In the first approach we tested each transcript against phenotype individually, using a simple linear regression (with the model Load = Expression) and assessing significance assuming the standard null distribution for the *F* statistic. In the second approach, we generated principal components from *a priori* subsets of transcripts, using the prcomp() function in R, and then assessed the correlation between the first principal component and bacterial load using a simple linear regression.

To correct for multiple testing, we used an false-discovery-rate (FDR) and/or Holm familywise error rate control approach, as described in the Results section, implemented using the p.adjust() function in R.

## Supporting Information

Figure S1 Power calculations for association tests. Power is calculated based on simulation as described in the Methods, assuming a minor allele frequency of either 0.25 or 0.50 and using estimates of variance components for the Gram-positive and Gram-negative bacteria included in this study.
(0.74 MB EPS)

Table S1 List of all markers with tests for association with bacterial load after infection with each of four bacteria (including sex by marker interaction tests).
(0.06 MB XLS)

Table S2 List of all gene-wise association tests.
(0.02 MB XLS)

Table S3 List of all genes represented on the BeadChip arrays, and their functional classification.
(0.05 MB XLS)

Table S4 List of all genotype-expression associations significant at a 10% false-discovery rate.
(0.07 MB XLS)

Table S5 Loading for expression of *E. faecalis* and *S. marcescens* induced effector genes onto the first principal component of effector expression.
(0.02 MB XLS)

Table S6 Annotation information for all SNPs included in this study.
(0.03 MB XLS)

Table S7 Genotypes for each line, and mean bacterial load after infection with each bacteria for all lines.
(0.21 MB XLS)

Table S8 Linkage disequilibrium for all marker pairs.
(0.33 MB XLS)

Dataset S1 Normalized BeadChip expression data after infection with either *Serratia marcescens* or *Enterococcus faecalis*.
(1.24 MB GZ)

Dataset S2 Raw BeadChip expression data.
(5.96 MB GZ)
